# Personality traits and pattern of beliefs of near-death(-like) experiencers

**DOI:** 10.3389/fnhum.2023.1124739

**Published:** 2023-04-28

**Authors:** Aminata Bicego, Héléna Cassol, Jessica Simon, Pauline Fritz, Simona Abagnale, Audrey Vanhaudenhuyse, Steven Laureys, Charlotte Martial

**Affiliations:** ^1^Sensation and Perception Research Group, GIGA-Consciousness, University of Liège, Liège, Belgium; ^2^Neurological and Functional Rehabilitation Center, University Hospital of Liège, Fraiture, Belgium; ^3^Coma Science Group, GIGA-Consciousness, University of Liège, Liège, Belgium; ^4^Psychology and Neuroscience of Cognition Research Unit (PsyNCog), University of Liège, Liège, Belgium; ^5^Centre du Cerveau^2^, University Hospital of Liège, Liège, Belgium; ^6^Interdisciplinary Algology Centre, University Hospital of Liège, Liège, Belgium; ^7^CERVO Brain Research Center, University of Laval, Québec, QC, Canada

**Keywords:** personality, near-death experience, near-death-like experience, Openness, Fantasy proneness, Spirituality

## Abstract

**Introduction:**

Little is known about the potential personality and psychological predictors of near-death experiences (NDEs), and fewer yet those of near-death-like experiences (NDEs-like; similar phenomenology reported after a non-life-threatening context). This study investigated whether personality traits (Openness, Extraversion, Pleasantness, Conscientiousness, and Neuroticism), dissociative experiences, Fantasy proneness, disposition toward auditory hallucinations, absorption trait, and endorsement of paranormal and spiritual beliefs could be associated with the recall of NDEs(-like).

**Methods:**

To this aim, we invited four groups of people to retrospectively fill in questionnaires assessing the following factors: NDE experiencers (*n* = 63), NDE(-like) experiencers (*n* = 31), controls with a life-threatening situation but no NDE(-like) (*n* = 43), and controls without a life-threatening situation or an NDE(-like) (*n* = 44). We carried out univariate analyses for each factor and then performed a multiple regression analysis and a discriminant analysis.

**Results:**

The multivariate logistic regression analysis revealed that the endorsement of spiritual beliefs was associated with the recall of NDEs-like while Openness and Fantasy proneness were associated with the recall of NDEs. The discriminant analysis showed that these variables produce 35% of correct classification.

**Discussion:**

Albeit retrospective, these results pave the way for future research on psychological predictors of NDEs(-like) by highlighting the influence of Spirituality, Openness, and Fantasy proneness on these phenomena.

## 1. Introduction

When people experience a near-death incident, disconnected consciousness can sometimes emerge in the form of dream-like experiences (Martial et al., 2020a). We call them *near-death experiences* (NDEs) if certain prototypical mystical features are present, such as out-of-body experiences, entering a gateway (e.g., a tunnel), and meeting entities (Martial et al., 2020b). In parallel, numerous subjective experiences closely resembling NDEs have been reported by people in various non-life-threatening situations, such as syncope (Lempert et al., 1994), consumption of recreational drugs (Martial et al., 2019), meditation (Van Gordon et al., 2018), and intense grief (Kelly, 2001). They have been termed “*near-death-like experiences*” (NDEs-like). Up to now, there is no empirical study indicating that the phenomenology of NDEs and NDEs-like differs (Charland-Verville et al., 2014).

In theory, every human could experience them, but empirical studies indicate an incidence of 10–23% in cardiac arrest survivors (van Lommel et al., 2001; Schwaninger et al., 2002; Greyson, 2003) and 4–8% in the general population (Knoblauch et al., 2001; Perera et al., 2005). Previous research on personality and cognitive profile showed that most individuals reporting an NDE do not present deficits in global cognitive functioning (Greyson, 2003) or specific pathological disorder (Gabbard and Twemlow, 1984; Greyson, 1997; Facco and Agrillo, 2012). Moreover, the occurrence of NDEs does not appear to be influenced by factors, such as educational level, social class, marital status (Wilson and Barber, 1982; Roberts and Owen, 1988; Zhi-ying and Jian-xun, 1992; van Lommel et al., 2001; Schwaninger et al., 2002; Greyson, 2003), state anxiety, neuroticism, or extroversion (Locke and Shontz, 1983). Nevertheless, the existing literature interviewing NDE(-like) experiencers (NDErs[-like]) suggests that some particular cognitive and personality factors may play a role in the generation (or, at least, the recall) of an NDE(-like). Notably, several types of research led by Greyson identified some variables, such as a higher proportion of purportedly psychic experiences (i.e., extrasensory perceptions, paranormal experiences such as *déjà vu*) (Greyson, 2003) and a tendency for (non-pathological) dissociative experiences (Greyson, 2000), in NDE experiencers (NDErs) compared with controls. More recently, Martial et al. (2018a) found a higher engagement in fantasy (i.e., Fantasy proneness) in NDErs-like as compared with control volunteers (without a life-threatening situation) but not in classical NDErs as compared with a control group of people who experienced a life-threatening situation without the recall of an NDE. In the same vein, Greyson and Liester (2004) found that in a sample of 73 interviewed NDErs, 80% reported auditory hallucinations (i.e., voices) after their NDE and 20% reported auditory hallucinations both before and after their NDE. They also noted that respondents reporting subsequent hallucinations are individuals who describe more elaborate NDEs [i.e., scoring very high on the Greyson NDE Scale (Greyson, 1983)]. In parallel, Twemlow and Gabbard (1985) found that NDErs scored higher on a measure of absorption [i.e., the propensity to focus attention on imaginative and selected sensory experiences to the exclusion of stimuli in the external environment (Tellegen and Atkinson, 1974)] than a group of people reporting “only” an out-of-body experience (Twemlow and Gabbard, 1985). More recently, a trend toward absorption predicting N,N-Dimethyltryptamine (DMT)-induced NDEs-like was demonstrated, however, the relationship did not reach significance (Timmermann et al., 2018).

A few authors have also been interested in religious beliefs in the NDE population, but the results do not show a clear link between religious orientation and NDEs' occurrence or intensity (Ring, 1980; Sabom, 1982). Indeed, when assessing whether religious orientation would influence the intensity of the NDE, Ring (1980) and Sabom (1982) could not find any convincing results. One study examined intrinsic religious beliefs in NDErs; nevertheless, no significant link between intrinsic religious beliefs and the intensity of the NDE (based on the narrative recall) was found (McLaughlin and Malony, 1984). Indirectly related to NDE, one study also found a positive correlation between endorsing paranormal beliefs, assessed by the Revised Paranormal Belief Scale (Tobacyk, 2004), and having lived an out-of-body experience (Tobacyk and Mitchell, 1987) or premonition (Tobacyk, 1991).

Only one study examined a varied range of personality traits (introversion–extraversion, sensing–intuition, thinking–feeling, judging–perceiving, and Fantasy proneness) and beliefs (paranormal and spiritual) in NDErs by comparing them with people who believed that NDE (who never experienced one) is proof of afterlife and controls (i.e., no NDE and no belief of an afterlife as NDE) (Gow et al., 2003). The results showed that NDErs scored significantly higher on scales assessing disposition toward fantasy, paranormal beliefs, and spiritual beliefs (e.g., “During altered states, such as sleep or trances, the spirit can leave the body”) as compared with controls but not people who believed that an NDE is proof of an afterlife (Gow et al., 2003). Nevertheless, this study has some limitations marked by a limited number of NDErs (*n* = 30), classification of participants into the different groups according to the subjective belief of participants (not Greyson NDE Scale's standardized cutoff), no inclusion of NDErs-like, and a correlational study design (Gow et al., 2003).

Altogether, previous research on personality and NDE(-like) has so far included only small sample sizes and no proper control groups (e.g., individuals who have experienced a life-threatening situation without an NDE). In addition, few studies assessed a single personality or cognitive trait in samples of NDErs, and only one study assessed various traits together. In the present study, we first looked at the different facets of the personality and beliefs of NDE(-like) experiencers by administering several standardized questionnaires, in comparison to two control groups. We aimed to explore whether any personality factors are consistently related to reporting NDE(-like). To this goal, we retrospectively compared personality traits in four groups: NDErs, NDErs-like, controls with a life-threatening situation but no NDE (controls w/LTS), and controls without a life-threatening situation (controls w/o LTS). Second, we determined whether personality differences or cognitive traits could predict membership in one of the four groups.

## 2. Materials and methods

### 2.1. Participants

NDErs(-like) were recruited from the NDErs database of the Coma Science Group (GIGA-Consciousness, University of Liège, Belgium) which includes 352 French-speaking people who claim to have experienced an NDE(-like), who gave their approval to be contacted again by providing their valid emails and/or postal addresses. All 352 people were invited to take part in the study. The presence of an NDE(-like) was identified and quantified using the Greyson NDE Scale [i.e., a cutoff score of ≥7/32; Greyson (1983)]. Participants from the control groups were recruited *via* announcements in local media. Recruitment and testing were in conformity with the local Ethics Committee of the Faculty of Medicine of the University of Liège. All participants signed informed consent in accordance with the Declaration of Helsinki and its later amendments.

### 2.2. Procedure

Participants were invited to take part in a study on personality *via* mailed questionnaires. It started with an introduction letter which contained all the information of the study, followed by informed consent. Participants were then invited to respond to sociodemographic items (i.e., sex, education, and age at the interview). For NDErs(-like), we asked information concerning the context of their experience as follows: age at NDE(-like), time since NDE(-like), presence of life-threatening situation and/or of coma (i.e., loss of consciousness >1h), as well as etiology (i.e., cardiac arrest, drowning, electrocution, brain trauma, vascular accident, anesthesia/surgery, sleep, other with a medical cause [e.g., sepsis], or other without a medical cause [e.g., syncope, orgasm]). The latest information allowed us to distinguish NDEs-like from classical NDEs. Participants in the control groups were asked whether they had ever experienced a life-threatening situation and/or a coma. To evaluate the occurrence of a life-threatening situation, we asked them to refer to which type of event they had gone through (see etiology categories) if they had a period of coma and the (approximate) date of the incident. This latter information allowed us to differentiate the two control groups. Subsequently, they had to answer a battery of seven questionnaires as follows: the Big Five Inventory Questionnaire (John and Srivastava, 1999); the Dissociative Experience Scale (Bernstein and Putnam, 1986); the Creative Experiences Questionnaire (Merckelbach et al., 2001); the Launay–Slade Hallucination Scale (Larøi et al., 2004); the Tellegen Absorption Scale (Tellegen and Atkinson, 1974); the Intrinsic Religious Motivation Scale (Hoge, 1972); and the Revised Paranormal Belief Scale (Tobacyk, 2004). For NDErs(-like), the Greyson NDE Scale (Greyson, 1983) was also administered before these seven questionnaires.

The participants in this study were classified into four groups, according to whether or not they had had an NDE based on the total score of the Greyson NDE Scale (Greyson, 1983) which was due to a life-threatening situation or not. The “NDErs” group includes people who experienced an NDE (i.e., reaching the cutoff score of ≥7/32 on the Greyson NDE Scale; Greyson, 1983) in a life-threatening situation. The “NDErs-like” group includes people who experienced an NDE-like (i.e., reaching the cutoff score of ≥7/32 on the Greyson NDE Scale; Greyson, 1983) out of a life-threatening situation. The “controls w/LTS” group includes people who lived a life-threatening situation but did not recall an NDE(-like). “Controls w/o LTS” includes people who had neither a life-threatening situation nor an NDE(-like).

### 2.3. Materials

The *Greyson NDE Scale* (Greyson, 1983) is composed of 16 questions that assess whether a person has had a subjective experience that can be considered an NDE. This scale is divided into four subscales (cognitive, e.g., “Did time seem to speed up?”; affective, e.g., “Did you have a feeling of peace or pleasantness?”; paranormal, e.g., “Were your senses more vivid than usual?”; and transcendental, e.g., “Did you seem to enter some other, unearthly world?”) and provides a maximum score of 32, with a score ranging from 0 to 2 (0 = not present, 1 = moderately or ambiguously present, and 2 = definitely present) for each question. A minimum score of 7 is the cutoff for a “true” NDE. It has good test–retest reliability and internal consistency and evaluates the richness of an NDE using a total score.

The *Big Five Inventory Questionnaire* (John and Srivastava, 1999) is a personality test that identifies five fundamental dimensions (Extraversion, e.g., “Is talkative”; Pleasantness, e.g., “Tends to find fault with others”; Conscience, e.g., “Does a thorough job”; Neuroticism, e.g., “Is depressed, blue”; and Openness, e.g., “Is original, comes up with new ideas”) for the description and evaluation of personality. It is composed of 45 items to which participants must respond using a five-point Likert scale (1 = strongly disagree, 2 = disagree a little, 3 = neither agree nor disagree, 4 = agree a little, and 5 = strongly agree) divided into five sections. Each section has a different score range (from a minimum of 8 to a maximum of 50). It is an economic, understandable, sufficiently exhaustive tool to describe the personality and can constitute a common and generalizable matrix in the evaluation process (Benjamin, 2002).

The *Dissociative Experience Scale* (Bernstein and Putnam, 1986) is a self-reported questionnaire used to evaluate the presence, quantity, and type of dissociative experiences one might live in daily life. It consists of 28 items (e.g., “Some people are so deep in thought that they don't hear the doorbell”) arranged on an analog scale (from 0 to 100% measuring the frequency of dissociative experiences), and the total score ranges from 0 to 100. There is a cutoff of 45 as a mean score to suggest a dissociative disorder. This questionnaire is not a diagnostic instrument; it is designed only for screening, but it can suggest that a specific clinical assessment is needed. The Dissociative Experience Scale is a valid and reliable tool for measuring dissociative experiences both in clinical samples and in control populations (Bernstein and Putnam, 1986; Steinberg et al., 1991; Carlson et al., 1993), revealing a similar factorial structure in groups of psychiatric patients and healthy subjects (Sanders and Green, 1994).

The *Creative Experiences Questionnaire* (Merckelbach et al., 2001) is a self-reported questionnaire that is composed of 25 true/false items (e.g., “As a child, I thought that the dolls, teddy bears, and stuffed animals that I played with were living creatures”) that measure Fantasy proneness. The items are referred to as the developmental antecedents, involvement, and consequences in fantasy and daydreaming. The total score range is 0–25. The higher the score, the higher the level of Fantasy proneness. This questionnaire has good internal consistency and optimal test–retest stability (Merckelbach et al., 2001).

The *Launey–Slade Hallucination Scale* validated the French version (Larøi et al., 2004) and aims to measure participants' hallucinations in terms of frequency, intensity level of control, and affective responses. It consists of 16 items (e.g., “I have had the feeling of touching something or being touched and then found that nothing or no one was there”) to which participants respond using a five-point Likert scale (0 = certainly does not apply to me, 1= possibly does not apply to me, 2 = unsure, 3 = possibly applies to me, and 4 = certainly applies to me). The total score range is between 0 and 64. A high score indicates a higher tendency to hallucinate. This scale has high reliability.

The *Tellegen Absorption Scale* (Tellegen and Atkinson, 1974) consists of 34 true/false items (e.g., “My thoughts often do not occur as words but as visual experiences”), aiming to measure the absorption of a person, meaning the disposition for having episodes of a deep involvement that engage all the subject's resources (perceptual, imaginative, and cognitive). The total score range is from 0 to 34. The higher the score, the more one has a high propensity for absorption.

The *Intrinsic Religious Motivation Scale* (Hoge, 1972) is a self-reported questionnaire that measures internal religious motivation. Internal religious motivation is considered intrinsic because it does not rely on external behavior but rather on the concept of religiosity itself. It consists of 10 items with a four-point Likert scale (1 = strongly disagree, 2 = disagree, 3 = agree, and 4 = strongly agree), with a total score range between 10 and 40. A high score indicates a high internal religious motivation. Some examples of the questions are as follows: “*My faith involves my whole life*”, “*One should seek God's guidance in making any important decision*”, and “*In my life, I experience the presence of the Divine”*.

The *Revised Paranormal Belief Scale* (Tobacyk, 2004) is a self-reported scale that gives a measure of belief in paranormal phenomena through seven dimensions as follows: Traditional Religious Belief (e.g., “The soul continues to exist though the body may die”), Psi [e.g., “Some individuals are able to levitate (lift) objects through mental forces”], Witchcraft (e.g., “Black magic really exists”), Superstition (e.g., “Black cats can bring bad luck”), Spiritualism [e.g., “Your mind or soul can leave your body and travel (astral projection)”], Extraordinary Life Forms (e.g., “The abominable snowman of Tibet exists”), and Precognition (e.g., “Astrology is a way to accurately predict the future”). It consists of 26 items to which participants respond using a seven-point Likert scale (1 = strongly disagree, 2 = moderately disagree, 3 = slightly disagree, 4 = uncertain, 5 = slightly agree, 6 = moderately agree, and 7 = strongly agree), with a total score ranging from 1 to 7. This scale has great internal reliability and good cross-cultural validity (Drinkwater et al., 2017). A high score indicates a high endorsement of paranormal beliefs.

### 2.4. Statistical analyses

First, descriptive statistics were conducted. Qualitative variables were expressed with counts and percentages. The normality of quantitative variables was examined graphically with histograms and quantile–quantile plots, and statistically by realizing a Shapiro–Wilk normality test. If normality was assumed for the distribution of the quantitative variable, mean and standard deviation (SD) were reported. Conversely, median and interquartile range were presented. Second, univariate analyses were performed to compare scores between the four groups (i.e., controls w/o LTS, controls w/ LTS, NDErs-like, and NDErs). The χ^2^ test was used in the case of categorical variables, and the one-way analysis of variance (ANOVA) or its non-parametric equivalent the Kruskal–Wallis test was used for the dimensions of the Big Five Questionnaire, the Dissociative Experience Scale, the Creative Experience Questionnaire, the Launey–Slade Hallucination Scale, the Tellegen Absorption Scale, the Intrinsic Religious Motivation Scale, and the Revised Paranormal Belief Scale. *Post-hoc* analyses were carried out with Tukey's test or its non-parametric equivalent the Dwass-Steel-Critchlow-Fligner pairwise comparison test. Cramer's V, eta square, and ε^2^ were used as a measure of effect size. Significant variables in the univariate case were included in a multivariate logistic regression to determine predictive variables associated with the occurrence of the recall of an NDE(-like) within a single statistical model. The dependent variables were the NDErs-like, NDErs, and controls w/LTS factors; the controls w/o LTS were taken as the referential category. Two-tailed *p*-values of < 0.05 were considered to be statistically significant. Then, we performed a discriminant analysis including only the variables identified in the multinomial logistic regression to test the classification function. Discriminant analysis is a multivariate technique used to separate two or more groups based on one or more linear combinations of selected variables, exploring the contribution of each variable in separating the groups defined before the study. Two-tailed *p*-values of < 0.05 were considered to be statistically significant. We also carried out a sensitivity power analysis (alpha = 0.01, a power of 0.80, total sample size = 181, and four groups). The acquired data were processed using statistical data processing software Jamovi 2.2.5^1^ and SAS 9.4 (© SAS Institute Inc.).

## 3. Results

### 3.1. Univariate analyses for participant characteristics

A total of 63 participants (35% out of the whole sample included in this study, i.e., total *N* = 181) reached the cutoff score for an NDE lived during a life-threatening situation, they formed the first group (“NDErs”). The second group was composed of 31 participants (17%) who had an NDE but were not related to a life-threatening situation (“NDErs-like”). The last two groups included control participants as follows: 43 participants (24%) lived a life-threatening situation but did not recall an NDE (“controls w/LTS”) and 44 participants (24%) had neither a life-threatening situation nor an NDE (“controls w/o LTS”). Considering sociodemographic data, there was no statistical difference in age at the NDE(-like) event, time since the NDE(-like) event, sex, education, and all subscales of the Greyson NDE Scale (Greyson, 1983) (*p* > 0.05) (Table 1). Significant differences were found for age at the interview (*p* < 0.001) with NDErs and NDErs-like being older than controls w/LTS and w/o LTS.

**Table 1:** Participant characteristics of NDErs, NDErs-like, control w/LTS, and control w/o LTS groups (total *n* = 181).

	**NDErs (*n =* 63)**	**NDErs-like (*n =* 31)**	**Controls w/LTS (*n =* 43)**	**Controls w/o LTS (*n =* 44)**	***p*-value**	**Effect size**
Age at interview,years, median (interquartile)	62 (15.0)	63 (18.5)	42 (23.5)	47.5 (27.3)	< 0.001	ε^2^ = 0.22
Age at NDE(-like),^¥^years, median (interquartile)	26 (24.0)	34 (25)	-	-	0.22	ε^2^ = 0.02
Time since NDE(-like),^¥^years, median (interquartile)	28 (22.5)	27 (29.5)	-	-	0.33	ε^2^ = 0.01
**Gender**, ***n*** **(%)**						
Female	32 (49)	21 (68)	22 (51)	29 (66)	0.22	Cramer's V = 0.16
Male	31 (51)	10 (32)	21 (48)	15 (34)		
**Education**, ***n*** **(%)**						
Elementary	1 (2)	0 (0)	0 (0)	0 (0)	0.85	Cramer's V = 0.11
High school	10 (16)	3 (10)	7 (16)	3 (7)		
Bachelor	19 (30)	9 (29)	16 (37)	15 (34)		
University	28 (44)	16 (51)	19 (45)	23 (52)		
PhD	5 (8)	3 (10)	1 (2)	3 (7)		
**Coma**, ***n*** **(%)**						
Yes	37 (59)	0 (0)	17 (39)	0 (0)	< 0.001	Cramer's V = 0.57
No	26 (41)	31 (100)	26 (61)	44 (100)		
Etiologies^*^, ***n*** **(%)**						
Cardiac arrest	9 (15)	0 (0)	2 (5)	-	< 0.001	Cramer's V = 0.70
Drowning	2 (3)	0 (0)	3 (8)			
Electrocution	2 (3)	0 (0)	0 (0)			
Brain Trauma	19 (30)	0 (0)	18 (46)			
Vascular accident	1 (2)	0 (0)	1 (2)			
Anesthesia/surgery	6 (9)	0 (0)	5 (13)			
Sleep	0 (0)	1 (3)	0 (0)			
Other w/medical cause	24 (38)	2 (7)	10 (26)			
Other w/o medical cause	0 (0)	28 (90)	0 (0)			
**Greyson NDE Scale** ^¥^						
Cognitive (0–32),median (interquartile)	3 (3)	3 (2)	-	-	0.26	ε^2^ = 0.01
Affective (0–32),median (interquartile)	5 (3.8)	6 (3.0)			0.59	ε^2^ = 0.00
Paranormal (0–32),median (interquartile)	4 (3.0)	4 (2)			0.5	ε^2^ = 0.00
Transcendental (0–32),median (interquartile)	3 (3)	4 (4)			0.47	ε^2^ = 0.01
Total,mean ± SD	15.7 (5.6)	16.2 (5.2)			0.69	η^2^ = 0.00

^*^Comparison was carried out between NDErs, NDErs-like, and Control w/ LTS. ^¥^Comparisons were carried out between NDErs and NDErs-like. NDErs, Near-Death Experiencers; NDErs-like, Near-Death-Like Experiencers; Controls w/LTS, Controls with a life-threatening situation; Controls w/o LTS, Controls without a life-threatening situation; SD, standard deviation; n, sample size.

### 3.2. Univariate analyses for personality and belief questionnaires

No statistical difference was found across groups for the Extraversion, Pleasantness, and Neuroticism subscales of the Big Five Inventory Questionnaire (John and Srivastava, 1999), the Dissociative Experience Scale (Bernstein and Putnam, 1986), the Launey–Slade Hallucination Scale (Larøi et al., 2004), and Superstition and Extraordinary life form subscales of the Revised Paranormal Belief Scale (Tobacyk, 2004) (Table 2). Significant differences were shown for the Openness and Conscientiousness subscales of the Big Five Inventory Questionnaire (John and Srivastava, 1999). Concerning the Openness subscale, controls w/o LTS were less open to experience than the NDErs-like, the NDErs, and the controls w/LTS (*p* = 0.01). While the Conscientiousness subscale was globally significant (*p* = 0.03), *post-hoc* analyses did not yield any significant differences between the groups. About the Creative Experience Questionnaire (Merckelbach et al., 2001), controls w/o LTS had significantly (*p* < 0.001) lower engagement in fantasy than NDErs, NDErs-like, and controls w/LTS. Concerning the Tellegen Absorption Scale (Tellegen and Atkinson, 1974), NDErs had a significantly (*p* = 0.004) higher disposition toward absorption than NDErs-like, controls w/LTS, and controls w/o LTS. Controls w/o LTS had significantly (*p* = 0.02) less intrinsic religious motivations than controls w/LTS, NDErs, and NDErs-like, assessed with the Intrinsic Religious Motivational Scale (Hoge, 1972). Finally, the groups significantly differed on various subscales of the Revised Paranormal Belief Scale (Tobacyk, 2004). NDErs had significantly more Traditional Religious Beliefs than NDErs-like, controls w/ LTS, and controls w/o LTS (*p* = 0.001). While the Psi subscale was globally significant (*p* = 0.02), *post-hoc* analyses did not yield any significant differences between the groups. The same pattern of results was found for the Witchcraft subscale (*p* = 0.2). For the Spiritualism subscale, NDErs-like scored significantly higher than NDErs, controls w/LTS, and controls w/o LTS (*p* < 0.001). Concerning the Precognition subscale, NDErs score significantly higher than NDErs-like, controls w/LTS, and controls w/o LTS (*p* < 0.001).

**Table 2:** Mean (SD) or median (interquartile ranges) for non-normal distribution of the 17 variables measured at the interview.

**Questionnaire (min-max)**	**NDErs (*n =* 63)**	**NDErs-like (*n =* 31)**	**Controls w/LTS (*n =* 43)**	**Controls w/o LTS (*n =* 44)**	***p*-value**	**Effect size**
**Big five questionnaire (8–50)**						
Extraversion	29 (8.5)	28 (8.0)	27 (8.5)	30 (10.0)	0.72	ε^2^ = 0.01
Pleasantness	42 (7.5)	43 (5.5)	42 (8.5)	42 (5.0)	0.47	ε^2^ = 0.01
Conscientiousness	37 (7.0)	38 (7.5)	35 (9.0)	35 (6.5)	0.03	ε^2^ = 0.05
Neuroticism	18 (8.5)	17 (13.5)	22 (10.5)	21 (12.3)	0.29	ε^2^ = 0.02
Openness	41 (6.0)^*^	42 (8.5)	40 (9.0)	37 (11.8)^*^	0.01	ε^2^ = 0.0715.5pt
**Dissociative experience scale (0–100)**	12.1 (15.0)	10.7 (17.9)	10.0 (16.8)	10.7 (9.2)	0.3	ε^2^ = 0.0215.5pt
**Creative experience questionnaire (0–25)**	8 (6.5)^*^	7 (7.0)^*^	6 (5.5)	4 (4.0)^*^	< 0.001	ε^2^ = 0.1215.5pt
**Launey-Slade Hallucination Scale (0–64)**	20 (18.0)	21 (25.0)	17 (12.5)	14 (19.3)	0.12	ε^2^ = 0.0315.5pt
**Tellegen absorption scale (0–34)**	19.0 (7.1)^*^	18.0 (7.2)	15.8 (1.0)	14.0 (6.8)^*^	0.004	η^2^ = 0.0215.5pt
**Intrinsic religious motivation scale (10–40)**	20 (8.0)^*^	21 (10.5)	18 (8.5)	16.5 (6.0)^*^	0.02	ε^2^ = 0.0615.5pt
**Revised paranormal belief scale (1–7)**						
Traditional religious belief	3.5 (2.3)^*^	3.3 (2.5)^*^	2.3 (2.3)	2.6 (2.0)^*^	0.001	ε^2^ = 0.09
Psi	3.8 (2.3)	4 (2.5)	2.5 (1.9)	3.0 (2.4)	0.02	ε^2^ = 0.05
Witchcraft	3.8 (2.8)	3.3 (3.3)	2 (2.8)	2.3 (2.8)	0.02	ε^2^ = 0.05
Superstition	1 (0.7)	1 (0.2)	1 (0.0)	1 (0.0)	0.45	ε^2^ = 0.01
Spiritualism	4.5 (2.6)^*^	5 (1.9)^*^	3 (3.9)	3 (2.1)^*^	< 0.001	ε^2^ = 0.16
Extraordinary life form	2.7 (1.3)	2.7 (2.0)	2 (1.0)	2 (1.1)	0.15	ε^2^ = 0.03
Precognition	3.5 (2.6)^*^	3.3 (2.0)^*^	1.8 (2.1)	2 (2.1)^*^	< 0.001	ε^2^ = 0.13

NDErs, Near-Death Experiencers; NDErs-like, Near-Death-Like Experiencers; Controls w/ LTS, Controls with a life-threatening situation; Controls w/o LTS, Controls without a life-threatening situation; SD, standard deviation; n, sample size; ^*^, *post-hoc* significant differences.

### 3.3. Multiple regression analysis

A 10-predictor logistic model was fitted to the data to test the research hypothesis regarding the likelihood of having factors that predict the likelihood of recalling an NDE (Table 3). Regarding the NDErs, two factors were significant predictors as follows: the Openness subscale of the Big Five Inventory Questionnaire (John and Srivastava, 1999) (*p* = 0.04) and Fantasy proneness [*p* = 0.02; assessed *via* the Creative Experience Questionnaire (Merckelbach et al., 2001)]. Concerning NDErs-like, only the Spiritualism subscale (*p* = 0.02) of the Revised Paranormal Belief Scale (Tobacyk, 2004) was a significant predictor, while the Fantasy proneness was a tendency (*p* = 0.05). No predictor was statistically significant for the controls w/LTS group.

**Table 3:** Results of multiple logistic regression analysis of potential predictors for NDErs: Tellegen Absorption Scale, Big Five Questionnaire (Conscientiousness and Openness subscales), Creative Experience Questionnaire, Intrinsic Religious Motivation Scale, and Revised Paranormal Belief Scale (Traditional Religious Belief, Psi, Witchcraft, Spiritualism, Precognition).

**Predictors**	**NDErs**		**NDErs–like**		**Controls w/LTS**	
	**(*****n** **=*** **63)**		**(*****n** **=*** **31)**		**(*****n** **=*** **43)**	
	**Estimate (95%CI)**	***p-*** **value**	**Estimate (95%CI)**	***p*** **-value**	**Estimate (95%CI)**	***p*** **-value**
**Tellegen absorption scale**	0.007 (−0.07–0.09)	0.86	−0.03 (−0.13–0.06)	0.51	−0.007 (−0.09–0.07)	0.33
**Big five questionnaire**						
Conscience	0.07 (−0.02–0.15)	0.13	0.07 (−0.04–0.17)	0.2	−0.01 (−0.09–0.06)	0.72
Openness	0.07 (0.002–0.32)	0.04	0.05 (−0.03–0.13)	0.24	0.03 (−0.03–0.09)	0.34
**Creative experience questionnaire/fantasy**	0.18 (0.03–0.32)	0.02	0.16 (−0.005–0.33)	0.05	0.13 (−0.02–0.29)	0.11
**proneness**						
**Intrinsic religious motivation scale**	0.02 (−0.08–0.13)	0.64	0.07 (−0.04–0.19)	0.23	0.06 (−0.04–0.17)	0.27
**Revised paranormal belief scale**						
Traditional religious belief	0.14 (−0.33–0.62)	0.55	0.02 (−0.51–0.55)	0.93	−0.14 (−0.64–0.35)	0.56
Psi	−0.20 (−0.63–0.23)	0.36	−0.12 (−0.62–0.39)	0.65	0.18 (−0.25–0.60)	0.42
Witchcraft	−0.11 (−0.49–0.26)	0.55	−0.28 (−0.74–0.17)	0.21	0.05 (−0.37–0.47)	0.81
Spiritualism	0.20 (−0.24–0.64)	0.37	0.63 (0.09–1.16)	0.02	−0.04 (−0.49–0.41)	0.86
Precognition	0.32 (−0.14–0.79)	0.17	0.14 (−0.39–0.67)	0.61	−0.27 (−0.77–0.24)	0.3

NDErs, Near-Death Experiencers; NDErs-like, Near-Death-Like Experiencers; Controls w/ LTS, Controls with a life-threatening situation; CI, confidence interval; n, sample size.

### 3.4. Discriminant analyses

Discriminant analysis including the 10 predictors identified earlier showed that the Openness subscale of the Big Five Inventory Questionnaire (John and Srivastava, 1999) and the Fantasy proneness (Merckelbach et al., 2001) and Spiritualism subscales of the Revised Paranormal Belief Scale (Tobacyk, 2004) can classify and predict the group membership of experiencers (Wilks'λ = 0.76, F_(9)_ = 5.49, *p* < 0.001, R^2^ = 0.24) but only for 35% of the sample. A low percentage of the controls w/o LTS (9%) and controls w/LTS (24%) groups was classified into the NDErs group. In contrast, a relatively higher percentage of NDErs-like (35%) was classified into the NDErs group (see Table 4).

**Table 4:** Classification results and error rates between the four groups of participants.

**Number of observations and percent classified into group**					
**From group**	**Controls w/LTS**	**Controls w/o LTS**	**NDErs**	**NDErs-Like**	**Total**
Controls w/LTS	12	18	4	9	43
	27.91%	41.86%	9.30%	20.93%	100%
Controls w/o LTS	15	18	3	8	44
	34.09%	40.91%	6.82%	18.18%	100%
NDErs	15	6	20	22	63
	23.81%	9.52%	31.75%	34.92%	100%
NDErs-like	3	6	8	14	31
	9.68%	19.35%	25.81%	45.16%	100%
Total	45	48	35	53	181
	24.86%	26.52%	19.34%	29.28%	100%
Priors	0.25	0.25	0.25	0.25	
**Error counts estimates for groups**					
Rate	0.7209	0.5909	0.6825	0.5484	0.6357
Priors	0.25	0.25	0.25	0.25	

NDErs, Near-Death Experiencers; NDErs-like, Near-Death-Like Experiencers; Controls w/ LTS, Controls with a life-threatening situation; Controls w/o LTS, Controls without a life-threatening situation.

## 4. Discussion

This study aimed to explore whether any personality factors are consistently related to reporting an NDE(-like) by retrospectively comparing personality traits and patterns of belief in four different groups (NDErs, NDErs-like, controls w/LTS, and controls w/o LTS) and carried out a multiple regression analysis to determine potential predictive factors. The results indicated that only the Spiritualism subscale of the Revised Paranormal Belief Scale (Tobacyk, 2004) was a significant predictor for NDErs-like, meaning that believing in astral journeys, reincarnation, and dissociation of mind and body, and that communicating with the dead are possible facts, may increase the likelihood of recalling an NDE outside of an LTS. It is worth mentioning that only three (out of 31) participants from the NDErs-like group experienced the NDE-like in the context of meditation or drug intake, while all the others reported their NDE-like in contexts where there was no willingness of inducing an altered state of consciousness permitting to experience an NDE-like. Spirituality, as a predictor, has been studied little in the context of NDEs. Only one study which used the same questionnaire as ours has indeed demonstrated a correlation between the presence of spiritual beliefs and NDEs, but this study was only correlational and did not include NDErs-like, preventing any comparison with our results (Gow et al., 2003). In parallel, it has been shown that the intensity of an NDE is strongly correlated with a change in spirituality after the experience (Greyson, 2006); people seem to change their perspective on life, endorse deep spiritual consciousness, and decrease their fear of death. One of the hypotheses for this change is related to the mystical aspect of the NDE (e.g., encounter with a mystical presence or being traveled to a mystical realm) (Greyson, 2006). The psychedelic literature shows that experiencing a mystical experience may provide an alternative perspective on the meaning of life that seems to account for the beneficial/therapeutic effects of psychedelic experience (Griffiths et al., 2006; Nicholas et al., 2018). Considering the fact that NDE and psychedelic experience can be highly similar in terms of phenomenology (Timmermann et al., 2018; Martial et al., 2019), one can hypothesize that they could share some similar psychological processes of change (Greyson, 2006).

Regarding the NDErs, two factors were significant predictors, such as the Openness subscale (John and Srivastava, 1999) and Fantasy proneness (Merckelbach et al., 2001). This means that being creative, open to new experiences, and having a high engagement in fantasy (e.g., daydreaming and vivid mental imagery) may increase the likelihood of recalling an NDE when confronted with an LTS. With respect to Openness, we are not aware of any studies that have examined this personality trait in the context of NDEs. However, the literature on other non-ordinary states of consciousness has shown that expert meditators score high on this trait (mindfulness meditation, van den Hurk et al., 2011; Zazen and Tai Chi meditation, Pokorski and Suchorzynska, 2018) and that psychedelic experience might lead to an increase in openness (Erritzoe et al., 2018). Congruently, greater openness to experience was related to more intense 3,4-methylenedioxymethamphetamine-induced altered states (Studerus et al., 2021), which could suggest a bidirectional link between openness and the phenomenology of a particular experience. Moreover, Openness is known to be negatively correlated with age. In our sample, the NDErs(-like) were older than the control groups, thereby highlighting that Openness effectively seems to be a determinant factor for people who have experienced an NDE. To note the Dissociative Experience Scale (Carlson et al., 1993), the Superstition, Precognition, and Traditional Paranormal Belief subscales of the Revised Paranormal Belief Scale (Tobacyk, 2004) are also affected by age (Ross, 1990; Lange et al., 2000). Nevertheless, none of these (sub) scales turned out to have a significant influence on NDE(-like) recollection. A limitation of the retrospective design of this study is that we do not have information on the participants' prior personality traits. Typically, personality traits are supposed to be (more or less) stable personality constructs, but as mentioned earlier, certain life experiences such as intense spiritual practice or psychedelic drug use can alter personality traits such as Openness. It is known that having an NDE is transformative and that people who experience it might score higher on this trait after the NDE (Greyson, 2006). Notably, the control groups are typical in terms of their scoring (Plaisant et al., 2005, 2010) to the Big Five Inventory Questionnaire (John and Srivastava, 1999). Regarding Fantasy proneness, as mentioned in the introduction, Martial et al. (2018a) demonstrated a positive correlation between this personality trait and NDErs-like, whereas this link did not reach significance in NDErs. The results of the present study indicate that NDErs and NDErs-like do score higher on this scale than the two control groups, but only for NDErs, the Fantasy proneness might be a predictor while it is a tendency for NDErs-like. Furthermore, we have found that NDErs(-like) only scored significantly higher than the three other groups (i.e., NDErs, controls w/o LTS, and controls w/LTS) on the Spirituality subscale of the Revised Paranormal Belief Scale (Tobacyk, 2004), which also turned out to be the only significant predictor of NDErs(-like) recollection. A hypothesis might be that the NDErs(-like) who endorse spiritual beliefs and/or practices (e.g., meditation) might be more inclined to either experience a particular experience in a spiritually-oriented manner (i.e., to attribute a spiritual dimension to an experience) or undergo an experience labeled as NDErs(-like) within the context of spiritual practice (e.g., meditation). These are slightly different but complementary results highlight the need for further investigation.

Importantly, the retrospective design of our study does not permit concluding any causal pathway, namely, whether NDErs(-like) occur more frequently in individuals with (previously established) high engagement in Openness, Fantasy, and Spirituality propensity or whether such experiences encourage Openness, Fantasy, and Spirituality propensities in individuals who were previously not prone to that. However, since the items of the questionnaires assess retrospective Openness, Fantasy proneness, and Spirituality, it is reasonable to hypothesize that high engagement in those factors, as a habitual tendency, makes people more likely to report subjective NDEs when exposed to certain physiological and/or psychological conditions. One can hypothesize that NDErs(-like) are particularly sensitive to episodes of disconnected consciousness and possess a special propensity to pick up subjective experiences that other people are blind to. Peinkhofer et al. (2021) recently raised an evolutionary hypothesis suggesting a specific biological benefit of the survival of NDE when facing a life-threatening situation. This would offer a less distressing “reality” when facing a potentially inescapable danger. However, it is worth noting that the fact to have experienced an NDE(-like) may have influenced the current personality of NDErs(-like) and potentially the way they answered the questionnaires administered in the present study.

In this study, we chose a series of questionnaires assessing some specific personality traits and some beliefs; however, it must be emphasized that personality traits and beliefs' patterns allowed us to correctly classify 35% of the sample. Notably, this highlights that personality-related variables might influence the occurrence and nature of NDEs. Indeed, cognitive factors such as memory characteristics can play a role. Recently, we demonstrated that NDErs seem more likely to have an illusory recollection of details associated with the generation of false memory (notably, this study did not assess the recall of NDE *per se*) (Martial et al., 2018b). Other factors such as those linked to the circumstances of the NDE(-like) itself might also play an important role.

The present study has several limitations. First, the study was retrospective, cross-sectional, and relied on self-report measures. Nevertheless, the sensitivity power analysis indicated 0.80 power to detect medium to high statistical effects (η^2^ = 0.08). Second, volunteers who participated likely represent a self-selected sample and consequently might not be representative due to a possible selection bias. Third, it would have been relevant to include more information such as the religion of the participants at the time of the NDE, whether they had dissociative experiences by other means (e.g., meditation and drugs), the presence of spiritual practices before the NDE, and medical information regarding the presence of a life-threatening context. Fourth, the procedure for the control groups and the NDE(-like) groups was not the same. Future studies should consider administering the exact same list of questionnaires to all the groups that are part of the study. Fifth, the NDErs(-like) and the control groups (both w/ and w/o LTS) have been recruited at different periods (although they did fill in the present questionnaire at the same period), and the NDErs(-like), as compared with the controls, had repeated experience in completing various questionnaires due to the fact that they are part of the Coma Science Group's database. Thus, this might have influenced the scoring of participants. Furthermore, having the scores on the NDE scale for the control groups would have been interesting. Sixth, our participants were Western, thereby limiting our ability to extrapolate the present results to other parts of the world. Future research should include a more heterogeneous sampling population by recruiting people from different cultural and religious backgrounds. Finally, there might be potential overlaps between items of the different scales, assessing different but closely related constructs. To better understand this phenomenon, prospective studies aiming at identifying potential predisposing factors (not limited to personality) to NDEs should be carried out. Nevertheless, those studies are very complex to conduct due to the spontaneous nature of an NDE(-like).

The results obtained do not allow us to draw any coherent conclusion on how to distinguish people who have experienced an NDE from those who have not. Nevertheless, this study is the first to explore a varied range of potential personality traits and beliefs that might predict, if not the occurrence, the likelihood to recall an NDE. Due to the transformative effect an NDE has on one's view of the world and self (Cassol et al., 2019), the rigorous study of potential predictors of an NDE is of great importance. Furthermore, Openness, Spirituality, and Fantasy proneness have been linked to NDE, and other non-ordinary states of consciousness such as mediation, psychedelics, and self-induced cognitive trance [i.e., a volitional (i.e., by will) non-ordinary state of consciousness, adapted from traditional Mongolian shamanic trance and abstracted from any ritual, spiritual, and cultural expression; (Flor-Henry et al., 2017; Gosseries et al., 2020; Grégoire et al., 2022)], paving the way for a knowledge base for future research. Indeed, new protocols assessing the personality characteristics and the phenomenological features of participants learning self-induced cognitive trance (a non-ordinary state of consciousness known to produce a phenomenology similar to that of NDEs) are currently being carried out. This will allow, among other things, a better understanding of the personality predisposition of NDE. Furthermore, prospective studies addressing the abovementioned limitations would provide a better understanding of the psychological mechanisms at play in this phenomenon.

## Data availability statement

The raw data supporting the conclusions of this article will be made available by the authors, without undue reservation.

## Ethics statement

The studies involving human participants were reviewed and approved by Ethics Committee of the Faculty of Medicine of the University of Liège (2013/292). The patients/participants provided their written informed consent to participate in this study.

## Author contributions

CM was the main investigator and designed the protocol. CM, HC, SA, and PF obtained the data. AB and JS analyzed the data. AB, JS, PF, and CM interpreted the data. AB and CM wrote the manuscript. All authors contributed to the revision of the manuscript. All authors were involved in editing the manuscript and approved the final version.

## Footnotes

^1^Jamovi - Stats. Open. Now. (no date). Available online at: https://www.jamovi.org/ (accessed September 9, 2022).
